# Survey Defining Lip Health—Dermatology and Health Care Providers' Perspectives

**DOI:** 10.1111/jocd.16636

**Published:** 2024-10-28

**Authors:** Ajay S. Dulai, Nabeel Ahmad, Nasima Afzal, Venita A. Sivamani, Hemali B. Gunt, Caitlin Egli, Raja K. Sivamani

**Affiliations:** ^1^ Integrative Skin Science and Research Sacramento California USA; ^2^ College of Medicine, University of Houston Houston Texas USA; ^3^ LearnHealth Inc. Sacramento California USA; ^4^ Burt's Bees Durham North Carolina USA; ^5^ College of Medicine St. George's University West Indies Grenada; ^6^ Pacific Skin Institute Sacramento California USA; ^7^ University of California Davis California USA; ^8^ School of Medicine Sacramento California USA; ^9^ College of Medicine California Northstate University Elk Grove California USA

**Keywords:** cheilitis, lip dryness, lip health, lip smoothness

## Abstract

**Background:**

There currently exists no consensus on objective features which are relevant in the assessment of overall lip health.

**Aims:**

This study seeks to identify features and factors that are associated with healthy lips. This survey will ultimately enable clinicians to objectively track lip health for clinical evaluations and clinical research.

**Methods:**

An anonymous survey was conducted among allied healthcare professionals in‐person and through email. The survey contained questions designed to assess the importance of features of lip health using a 5‐point Likert scale. Features included lack of lip dryness; even tone or lack of hyperpigmentation; lip smoothness; lip color; lip shine, radiance, or luster; definition of the vermillion border/defined lip contour; lip fullness or plumpness; lack of lip lines; and lip firmness. The final questions prompted respondents to select the top three most important features when assessing lip health and to provide any additional pertinent factors.

**Results:**

A total of 334 responses were received, including board‐certified dermatologists (35.9%), dermatology residents (14.7%), and other healthcare specialties (49.4%). The features most cited were lack of lip dryness (65.8%), lip smoothness (38.4%), even tone or lack of hyperpigmentation (38.1%), definition of the vermillion border/defined lip contour (28.0%). Survey results reveal that lip dryness, lip smoothness, lack of hyperpigmentation, and definition of the vermillion border/defined lip contour were the features most associated with lip health.

**Conclusion:**

The results from this survey will provide a basis for future research in the field of lip health, research, and clinical treatment.

## 1 Introduction

While there exists a heavy focus on lip cosmetics, there is currently a lack of research into the health of the lips. Even though the lips are involved in dermatoses such as actinic cheilitis, lip photoaging, and oral lichen planus, there remains no consensus on the features of the lip which are relevant to its health. This study seeks to gain insight into the perspectives of dermatologists, dermatology residents, and other health practitioners on this topic. Identifying the most important features of healthy lips will contribute to the creation of a standardized assessment which has the potential to improve research and clinical practice.

**FIGURE 1:**
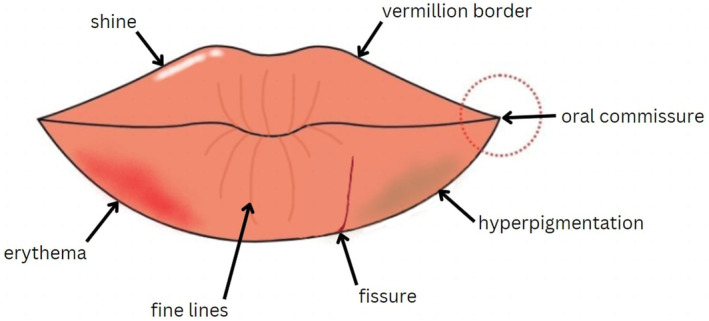
Lip diagram demonstrating some of the features that have been previously used to assess lip health.

Currently, three publications identified features of lips which are relevant to the assessment of lip health. One study developed a photo numeric lip health assessment scale involving three anatomical features (Figure 1): lip shine, texture, and vermillion border [1]. While this scale achieved 100% consensus from three board‐certified dermatologists, the 103 included subjects only included Fitzpatrick skin types I–III. A second study identified dryness and roughness, fine lip lines, lip texture, hydration, and barrier function as features in South Asian women (Fitzpatrick skin types IV–VI) as indicators of overall lip health [2]. Finally a third study created an objective scale to assess isotretinoin‐associated cheilitis [3]. The features included in this scale include erythema, scale/crust, fissures, and inflammation of the commissures.

To gain further insight into healthy lips, we created a survey for healthcare practitioners to evaluate the importance of various features when assessing lip health.

## 2 Methods

This study received approval from Institutional Review Board prior to initiation. The survey was administered anonymously to healthcare practitioners via SurveyMonkey and consisted of 13 questions. Participants were recruited through the LearnHealth network of practitioners, the 2023 Integrative Dermatology Symposium, and through online contact. Participants were asked to rate the importance of the following characteristics on a 5‐point Likert scale: lack of lip dryness; lip smoothness; even tone or lack of hyperpigmentation; definition of the vermillion border/defined lip contour; lip color; lip shine or radiance or luster; lack of lip lines; lip fullness/plumpness; lip firmness. Next, survey respondents were asked to provide their opinion on the top three most important features that define lip health. Finally, the respondents were provided an open‐ended question to provide any additional characteristics that were not included in the list.

## 3 Results

**FIGURE 2:**
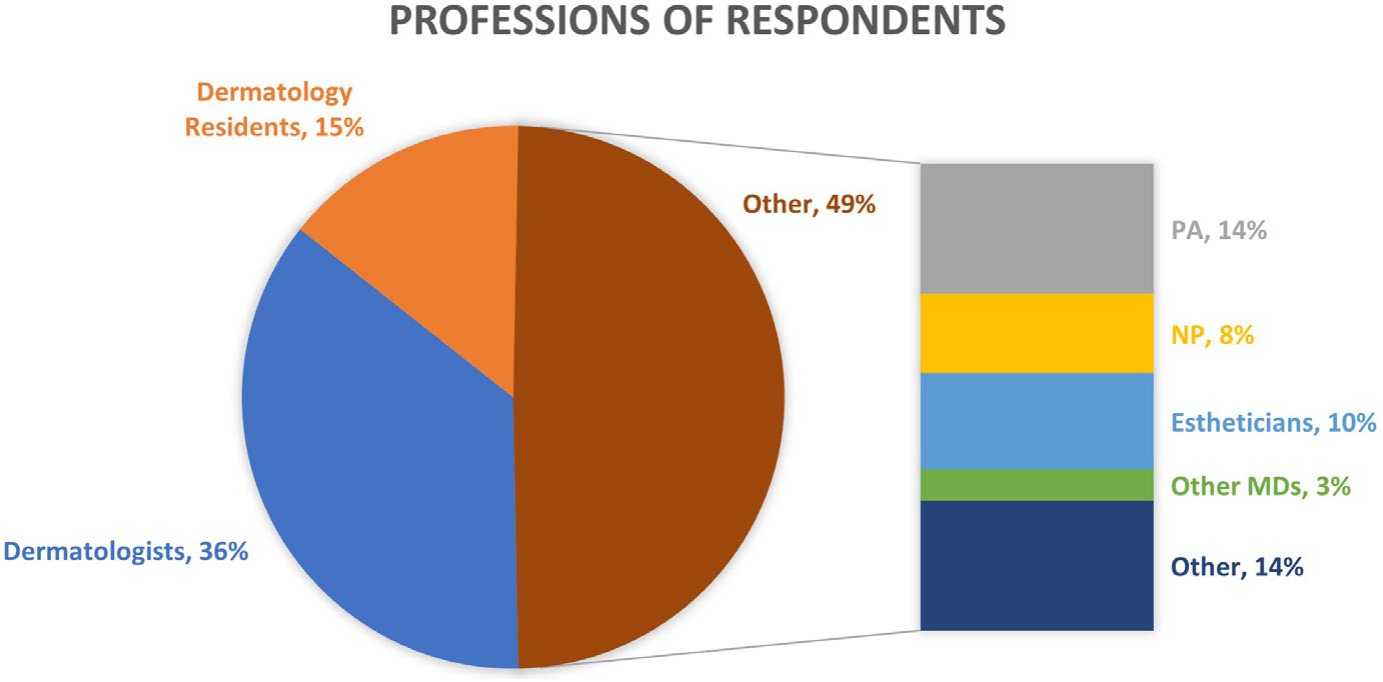
Composition of the professions of survey respondents.

The lip health survey gathered responses from 334 participants located in the USA, including 169 dermatologists and dermatology residents, 46 physician assistants (PA), 28 nurse practitioners (NP), and 91 other allied health professionals (including estheticians and other MDs; Figure 2).

Overall, the survey respondents rated the characteristics in the following order: lack of lip dryness; lip smoothness; even tone or lack of hyperpigmentation; definition of the vermillion border/defined lip contour; lip color; lip shine or radiance or luster; lack of lip lines; lip fullness/plumpness; lip firmness.

**FIGURE 3:**
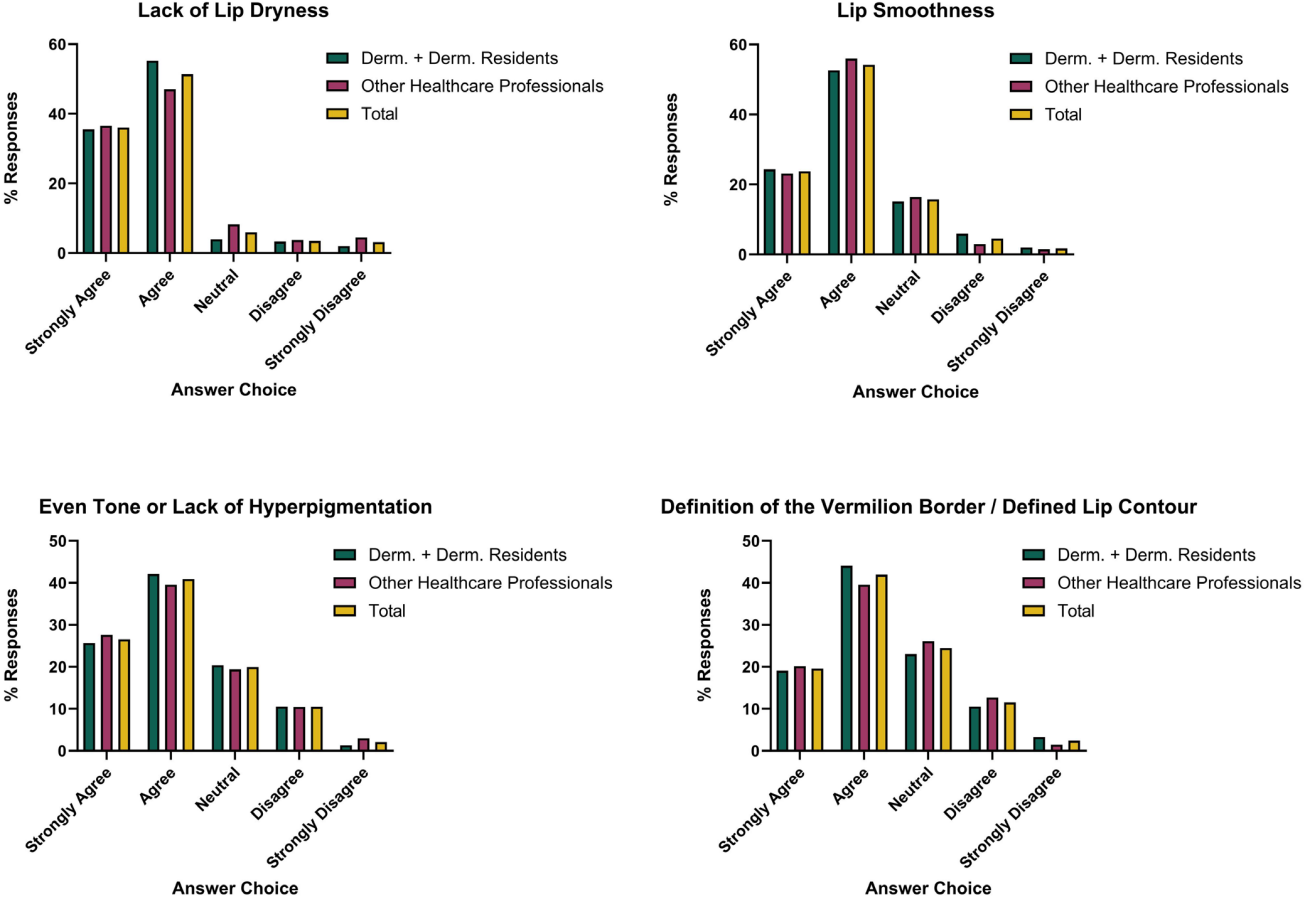
This figure displays the proportions of responses of the top 4 overall most important visual features of healthy lips.

**FIGURE 4:**
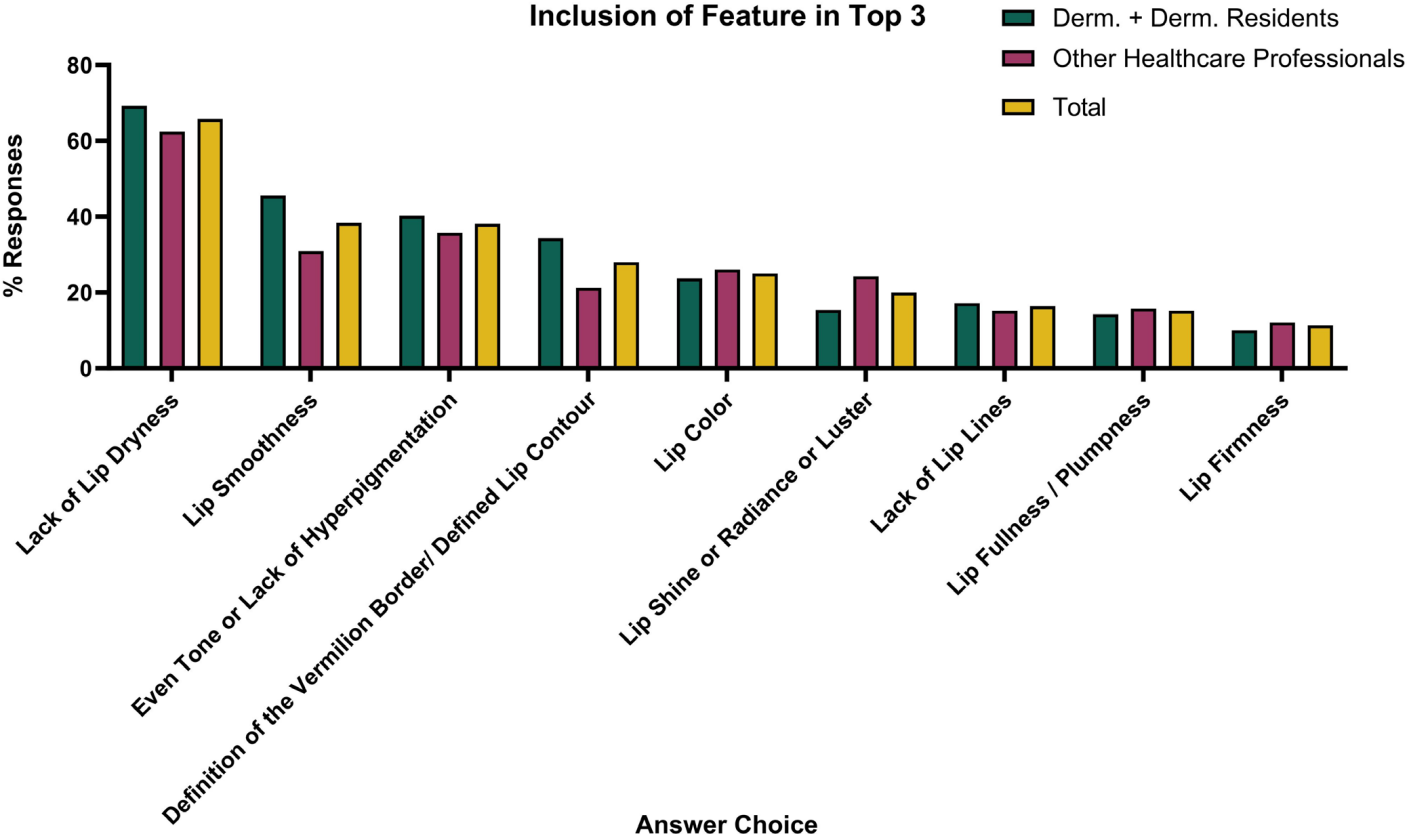
Proportion of responses which included the features in the top 3 most important visual characteristics of healthy lips.

The top three most important visual features included lack of lip dryness (65.8%), lip smoothness (38.4%), and even tone or lack of hyperpigmentation (38.1%; Figure 3). There was no change in the top three features when stratifying results of dermatologists against other professions. Overall, 87.4% of respondents strongly agreed (36%) or agreed (51.4%) that lack of lip dryness was an important feature of healthy lips. Furthermore, 78% and 60.5% of respondents strongly agreed (23.8%, 19.6%) or agreed (54.2%, 40.9%) that lip smoothness and even tone or lack of hyperpigmentation, respectively, were key features (Figure 4).

The fourth most relevant feature chosen both by dermatologists/dermatologist residents and overall was definition of the vermillion border/defined lip contour (34.3%). Whereas the fourth feature chosen by other healthcare professionals was lip color (26.1%).

Common additional characteristics reported from the open ended question were similar across healthcare specialties. Lack of lip lesions (19), state of oral commissures (7), lack of cheilitis (8), and lack of burning sensations (8) were the most frequently reported additional characteristics by both dermatologists and other healthcare professionals.

## 4 Discussion

Lack of lip dryness was rated to be the most important visual feature of healthy lips. Lips are susceptible to dryness due to the thin layer of stratum corneum. This leads to poor barrier function, decreased hydration, and up to three times increase in transepidermal water loss (TEWL) compared to the skin of the cheek [4, 5]. Factors that can contribute to increased lip dryness include younger age [6] and cold weather [7]. This is a relevant characteristic of patient quality of life due to resultant pain and irritation.

Lip smoothness has been correlated with increased conductance and increased levels of the ceramides CER[NH], CER[NP], CER[AH], CER[EOS], and CER[EOH] [8]. Lip conductance has been determined to be one of the factors associated with skin hydration [9]. Additionally, increased prevalence of ceramides CER[NP] and CER[NH] have been correlated with improvement of dry skin symptoms [10].

Even tone or lack of hyperpigmentation was the final feature included in the top three. This feature is clinically relevant due to conditions such as solar lentigines (a hallmark of photoaging) and mucosal melanoma. Additionally, assessment of the tone of lips can provide insight into the degree of UV damage [11].

Definition of the vermillion border/lip contour was the fourth most relevant feature chosen by dermatologists and dermatology residents. The vermillion border can be affected by granulomatous rosacea [12] and is the most common site of actinic cheilitis [13]. The fourth feature selected by other allied health professionals included lip color. Lip color is highly variable between ethnicities [14] and is difficult to associate with clinical pathology. For this reason, assessment of even tone or lack of hyperpigmentation may provide more useful clinical relevancy.

In this study commonly reported additional characteristics of healthy lips included lack of lip lesions, lack of cheilitis, and state of oral commissures. Common lip lesions include herpetic lesions which are caused by HSV‐1. Cheilitis can describe inflammation from various etiologies. Actinic cheilitis poses the most malignant threat due to the precancerous potential to become squamous cell carcinoma [15]. Angular cheilitis is a condition that affects the oral commissures and is a commonly impacted area of irritation.

A limitation of this study is that this is a survey study and future studies should incorporate our findings to assess whether these characteristics can be tracked in interventional clinical studies. The gender and the age of the survey respondents were not collected and the survey responses could not be sub‐analyzed based on the participant's demographics such as gender, or age. Future studies should incorporate these measures to allow for more sub‐stratification of the data to assess for their influence on the perception of lip health.

## 5 Conclusion

The results from this first‐of‐its‐kind survey provide us insight into how healthcare practitioners evaluate healthy lips. The top four most important visual features of healthy lips, as rated across all professions, include lack of lip dryness, lip smoothness, even tone or lack of hyperpigmentation, and definition of the vermillion border/defined lip contour. These features have clinical significance and are associated with various dermatoses. Future work in this area of research should consist of the creation of a photonumeric grading scale involving these top features. Ultimately, this will provide objectivity and consistency for clinical practice and research.

## Author Contributions

**Ajay S. Dulai:** drafting of protocol, collecting survey responses, analysis, writing of manuscript. **Nabeel Ahmad:** collecting survey responses, editing of manuscript. **Nasima Afzal:** collecting survey responses, drafting of protocol. **Venita A. Sivamani:** conception of idea, collecting survey responses, editing manuscript. **Hemali B. Gunt:** conception of idea, editing manuscript. **Caitlin Egli:** editing manuscript, drafting figures. **Raja K. Sivamani:** conception of idea, editing of manuscript.

## Ethics Statement

This study was approved by the Allendale IRB on 09/21/2023. Subject consents were collected at the beginning of the survey.

## Conflicts of Interest

R.K.S. serves as a scientific advisor for LearnHealth, Codex Labs, and Arbonne and as a consultant to Burt's Bees, Novozymes, Nutrafol, Novartis, Bristol Myers Squibb, Abbvie, Leo, Almirall, UCB, Incyte, Pfizer, Sanofi, Novartis, Sun, and Regeneron Pharmaceuticals. H.B.G. serves as the head of clinical and scientific affairs at Burt's Bees.

## Data Availability

The data that support the findings of this study are available from the corresponding author upon reasonable request.
